# Remodeling of inhibitory synaptic connections in developing ferret visual cortex

**DOI:** 10.1186/1749-8104-5-5

**Published:** 2010-02-01

**Authors:** Matthew B Dalva

**Affiliations:** 1 Department of Neuroscience, BRB II/III rm 1114, University of Pennsylvania Medical School, 421 Curie Blvd, Philadelphia, PA 19104, USA

## Abstract

**Background:**

In the visual cortex, as in many other regions of the developing brain, excitatory synaptic connections undergo substantial remodeling during development. While evidence suggests that local inhibitory synapses may behave similarly, the extent and mechanisms that mediate remodeling of inhibitory connections are not well understood.

**Results:**

Using scanning laser photostimulation in slices of developing ferret visual cortex, we assessed the overall patterns of developing inhibitory and excitatory synaptic connections converging onto individual neurons. Inhibitory synaptic inputs onto pyramidal neurons in cortical layers 2 and 3 were already present as early as postnatal day 20, well before eye opening, and originated from regions close to the recorded neurons. During the ensuing 2 weeks, the numbers of synaptic inputs increased, with the numbers of inhibitory (and excitatory) synaptic inputs peaking near the time of eye opening. The pattern of inhibitory inputs refined rapidly prior to the refinement of excitatory inputs. By uncaging the neurotransmtter GABA in brain slices from animals of different ages, we find that this rapid refinement correlated with a loss of excitatory activity by GABA.

**Conclusion:**

Inhibitory synapses, like excitatory synapses, undergo significant postnatal remodeling. The time course of the remodeling of inhibitory connections correlates with the emergence of orientation tuning in the visual cortex, implicating these rearrangements in the genesis of adult cortical response properties.

## Background

Inhibitory circuitry in the cortex is generated through a diverse array of specific types of inhibitory neurons. Local inhibitory interactions target specific regions of neurons and act in particular layers of cortex [1]. Generation of these specific connections relies on molecular cues and neuronal activity [2,3]. Importantly, the emergence of the adult pattern of GABAergic connections has been shown to correlate with periods of cortical plasticity and the development of mature cortical response properties [4]. However, less is known about the development of the inhibitory inputs onto excitatory neurons within cortical layers.

Pyramidal neurons form roughly 80% of neurons in layers 2/3 and 5 and send long axon collaterals horizontally to interconnect functionally similar domains. During development of layers 2 and 3 of primary visual cortex, an initially crude pattern of horizontal connections gives rise to a lattice of intrinsic excitatory connections that specifically links iso-orientation columns [5-9]. The development of excitatory axonal projections involves both local axonal branching and retraction of existing collaterals; these events are at least partially regulated by neuronal activity [9-12] and result in a period of exuberant synaptic inputs that are refined to generate the mature pattern of connectivity. Once the brain matures, the refined long-range horizontal connections link regions with similar response properties likely mediating extra-receptive field effects on unit responses [13].

Inhibitory interneurons in layers 2 and 3 comprise a diverse class of cells with specific patterns of local axonal arborizations and connections [3,14,15]. Anatomical evidence suggests that development of the connections of at least one class of interneurons - large basket cells - may involve elaboration and retraction of their axonal processes [6,7,16,17]. While mapping experiments suggest that inhibitory connections undergo refinement [18], how the mature pattern of inhibitory inputs made onto excitatory cells within visual cortex emerges is not well understood.

One mechanism that may contribute to the formation of the mature pattern of inhibitory inputs is the ability of gamma-aminobutyric acid (GABA) to act as an excitatory neurotransmitter early in development [19]. In a number of systems the excitatory activity of GABA appears to be a critical component for activity-dependent development of the mature pattern of inhibitory connections. However, the role of GABA excitation in the maturation of intralaminar cortical inhibitory connections is not well described.

To examine the mechanisms underlying the development and rearrangement of functional inhibitory synaptic connections, we used scanning laser photostimulation in tangential brain slices of ferret visual cortex to map the patterns of inhibitory and excitatory inputs within layer 2/3 onto single neurons at different times during development. By distinguishing excitatory and inhibitory inputs, we find that both types of local connections undergo developmental remodeling. Refinement of inhibitory connections to a mature pattern occurs before the refinement of excitatory connections and is correlated with the timing of the emergence of direction selectivity. Building on findings that remodeling of GABAergic connectivity may, in part, rely on GABA acting as an excitatory neurotransmitter, we used photostimulation to uncage GABA and explore when during development it acted as an excitatory transmitter. Consistent with previous reports in many developing systems, during early development GABA uncaging causes excitation. Interestingly, the change from excitatory to inhibitory transmitter by GABA correlates with a period of rapid refinement in inhibitory inputs. Taken together, these findings provide a detailed description of the maturation of excitatory and inhibitory inputs in the super granular layers of ferret visual cortex.

## Results

**Figure 1:**
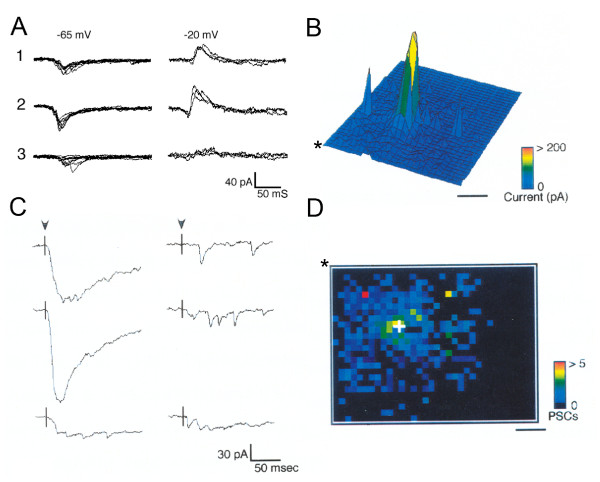
**Methods for visualizing synaptic inputs using photostimulation**. **(A) **Inhibitory and excitatory inputs can be distinguished by shifting the holding potential of the recorded neuron. Traces from photostimulation of three different sites shown with holding potential set at -65 mv (left) or -20 mv (right). Each site is stimulated several times at each holding potential to illustrate the stereotyped response to repeated photostimulation. Site one is scored as inhibitory only. Site two is scored as excitatory and inhibitory. Site three is scored as excitatory only. **(B) **Amplitude plot of a map of 982 stimulation sites covering an area of 2.0 mm^2 ^in a tangential slice of young (postnatal day 30) ferret visual cortex. The amplitude of the response at each site is coded by the color and height of the peak. The large central peak is due to direct activation of glutamate receptors on the recorded neuron. **(C) **Traces from stimulation of a few sites in the map shown in (B). On the left are traces from near to the recorded neuron containing direct and synaptic currents. Evoked synaptic currents are typically of similar amplitude but can vary in number. On the right are traces from stimulation of sites distant from the recorded neuron. Many stimulated locations do not generate a direct or synaptic response. **(D) **Plot of the number of synaptic currents evoked by stimulating each site. The post-synaptic current (PSC) plot shown is the result of analyzing the map in (B). In (D) the map is rotated and the three-dimensional perspective removed relative to (B). The asterisk is located at the same corner of the maps in (B) and (D). This plot enables the patterns and locations of inputs to be easily discerned. Scale bars = 500 μm.

**Figure 2:**
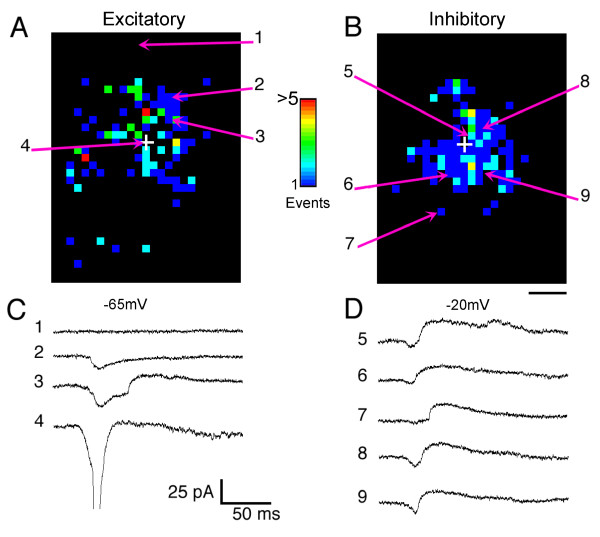
**Maps of inhibitory and excitatory inputs are distinct**. **(A) **Map of the locations and number of excitatory inputs to a pyramidal neuron (P58) evoked by photostimulation in a tangential slice of ferret visual cortex. Excitatory inputs arise from sites local (>300 μm) to and at longer distances from the cell body (indicated by the white cross). **(B) **Map of inhibitory inputs to the same neuron. Inhibitory inputs arise primarily within 300 μm of the cell body. **(C, D) **Electrophysiological recordings from sites indicated by the numbered arrows in (A, B). Photostimulation at the cell body evoked an action potential (4). Stimulation of other sites evoked excitatory (2, 3), inhibitory (7), or both excitatory and inhibitory inputs (3, 5, 6, 8, 9). To obtain the maps shown here, 1,467 locations were stimulated, mapping an area of approximately 2.1 mm^2^. Scale bar = 250 μm.

A total of 76 cells, at different postnatal ages, were analyzed using glutamate uncaging and photostimulation (Table 1). Here the characteristics of the maps of synaptic inputs onto these neurons are compared. Inhibitory and excitatory currents were distinguished by recording from neurons at different holding potentials. An experiment typically consisted of photostimulating the same array of locations surrounding the recorded neuron at two different holding potentials. At -65 mV holding potential inhibitory inputs were small due to the weak driving force for chloride ions, and generated downward deflections of the recording trace. At -20 mV holding potential (range -30 mV to -10 mV) inhibitory inputs reversed and produced upward deflections, but excitatory currents still produced small downward deflections of the trace (Figure 1A). Maps of the locations of evoked synaptic currents were generated to provide a clear representation of the pattern of synaptic inputs to each recorded neuron (Figure 1B-D). Photostimulation mapping of the inputs to single neurons at -65 mV and -20 mV holding potentials revealed distinct patterns of inputs (Figure 2). Occasionally, single passes were made at -20 mV, a potential at which inhibitory and excitatory currents were detectable simultaneously. At all ages, the size of the area stimulated and the actual number of sites stimulated per cell were comparable (Table 1).

**Table 1:** Summary of the data set on which this report is based

Group	N	Stimulated sites	Area (mm^2^)	EPSCs	IPSCs
P20 to P22	7	921.3 ± 168.0	1.41 ± 0.28	185.4 ± 40.6	136.7 ± 6.7
P30 to P35	9	1,030.2 ± 155.3	1.78 ± 0.19	363.7 ± 69.6	338.0 ± 68.3
P36 to P40	19	1,133.3 ± 115.4	1.81 ± 0.29	278.4 ± 45.8	213.0 ± 29.7
Mature	29	920.3 ± 137.9	1.47 ± 0.24	113.2 ± 14.1	76.8 ± 12.5
					
Total	64	1,001 ± 49.1	1.61 ± 0.12	235.2 ± 42.5	191.1 ± 29.3

The average number of locations stimulated, and the average area encompassing those sites, were similar for all ages, but the numbers of sites that when stimulated evoked excitatory post-synaptic currents EPSCs and inhibitory post-synaptic current (IPSCs) changed significantly. All numbers are means ± standard error of the mean.

### Inhibitory synapses remodel during postnatal cortical development

**Figure 3:**
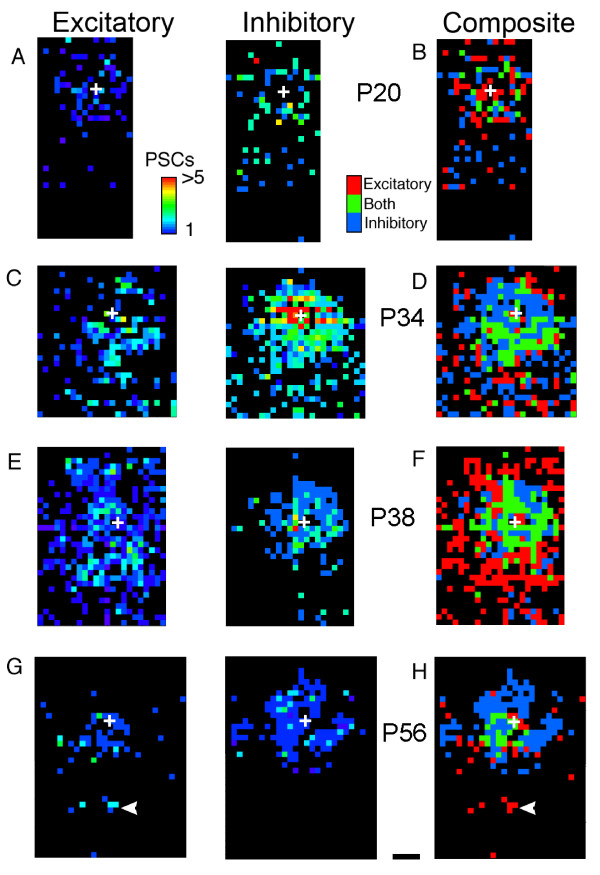
**Changing patterns of inhibitory inputs and relationships between inhibitory and excitatory inputs at different postnatal ages**. **(A) **Map of inhibitory inputs onto a P20 pyramidal neuron. The number of inputs is represented by the colored squares (scale bar at right); black indicates that no inputs were generated at that location (same scale applies for (C, E, G)). Relatively few sites generate inputs to this cell, and all are located within 500 μm of the cell body (indicated by the white cross). We stimulated 644 sites, mapping an area of approximately 1 mm^2^. PSC, post-synaptic current. **(B) **Map of the same cell in (A), but indicating sites that, when stimulated, generated excitatory (red), inhibitory (blue), or both (green) types of synaptic inputs (same convention applies to (B, D, F, H)). The number of events is not coded in these plots. Excitation dominates locally, as indicated by the red regions close to the cell. Inhibition dominates with increasing distance as indicated by the abundance of blue squares. **(C) **Example of the pattern of inhibitory inputs onto a P34 pyramidal neuron. Both the number of inputs and the area from which they arise have increased substantially compared to P20 to 22. The map consisted of 1,174 points covering 1.7 mm^2^. **(D) **At P34, inhibitory inputs predominate; close to the cell body stimulation evokes a mixture of excitatory and inhibitory inputs whereas at earlier ages (B) excitation dominated. **(E) **Map of inhibitory inputs into a P38 pyramidal neuron from 1,029 stimulation sites, covering an area of 1.4 mm^2^. At this age, the overall number of sites generating inhibitory inputs has declined; the decline is especially pronounced at longer ranges (>500 μm). **(F) **At P38, the pattern of excitatory and inhibitory inputs is also markedly different. Fewer sites generate exclusively inhibitory inputs, and longer range inputs are exclusively excitatory. **(G) **Map of a mature (P56) pyramidal neuron (1,019 points covering 2.3 mm^2^). The pattern of inhibitory inputs is similar to that seen at P38: most inhibitory post-synaptic currents are generated close to the neuron cell body. **(H) **The plot of excitatory and inhibitory inputs reveals significant changes in the pattern of excitatory inputs: far fewer are present than at P38 and those that are form occasional clusters (arrowhead). Scale bar (A-H), 250 μm.

**Figure 4:**
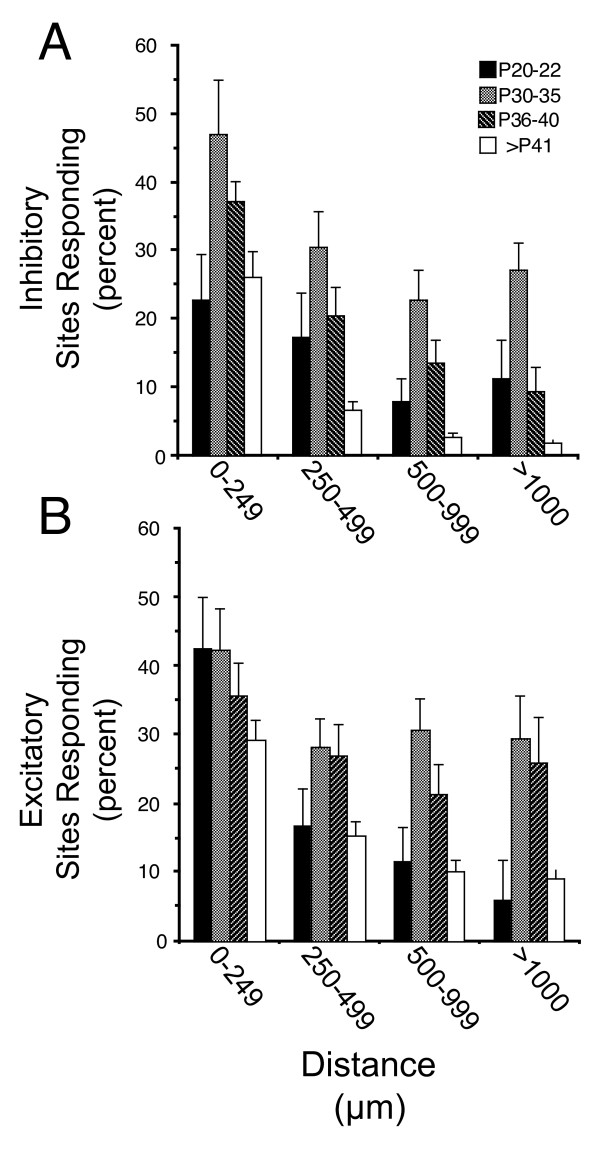
**The distribution of inhibitory and excitatory inputs shifts with developmental stage**. **(A) **The average percentage of stimulated sites that generated inhibitory post-synaptic currents (IPSCs) at various distances from the cell body for each age group. At all distances, the percentage of responding sites increased between P20 and P22 and P30 and P35, after which the percentages at all distances declined. The declines were most pronounced at greater lateral distances from the soma, implying a preferential elimination of longer-range inhibitory inputs. **(B) **Age-related changes in the origins of excitatory post-synaptic currents (EPSCs) with distance from the cell body. The average percentage of stimulated sites that generated EPSCs at different ages is generally similar to the pattern seen with IPSCs (A). The major difference is the retention of a significant number of inputs originating at >500 μm from cell bodies; these probably reflect long-range horizontal connections. Error bars = standard error of the mean.

At all ages inhibitory inputs originated from within 1.5 mm of the recorded neurons. Soon after layer 2/3 neurons have migrated into place (postnatal day (P)20 to P22), inhibitory synaptic inputs onto each recorded neuron could be evoked by photostimulation at numerous sites (n = 7 pyramidal cells; Table 1). We quantified the patterns observed in our mapping experiments by determining the number of locations that generated a specific type of response. We provide examples of typical photostimulation maps from each age group examined in Figure 3 and quantification across all neurons mapped in Figure 4. For neurons in brain slices of young animals (P20 to P30), most inputs arose from locations within 500 μm of recorded neurons (82.7 ± 6.7%; Figures 3A and 4A). By eye opening (P30 to P35 in ferrets) sites generating inhibitory post-synaptic currents (IPSCs) were far more numerous (n = 7; Table 1, Figure 4A). Within 250 μm of cell bodies, the proportion of locales evoking inhibitory inputs doubled between P20 and P22 and P30 and P35 (from 23 ± 7% to 47 ± 8%, *t*-test, *P *< 0.02). Similar increases were seen at all distances greater than 250 μm (Figure 4A; *t*-test, *P *< 0.04). In both age groups the density of inputs declined with increasing distance from the recorded neuron (Figure 3C).

The number and spatial extent of inhibitory inputs peaked at P30 to P35. During the following days (P36 to P40), the percentage of stimulated sites that evoked inhibitory inputs at distances greater than 250 μm from the recorded neurons declined by half (from 27 ± 3% at P30 to P35 to 14 ± 2% at P36 to P40; n = 8; Figure 3D versus Figure 3A; *t*-test, *P *< 0.0002). Within 250 μm of the cell body, the decline in sites evoking IPSCs was not significant (47 ± 8% to 37 ± 3%; Figure 3A; *t*-test, *P *> 0.18). Thus, during the period immediately after eye opening a significant fraction of the inhibitory synapses onto cells disappears (Table 1), implying that the extent of their individual axon arbors is reduced or that the specificity of connectivity increases. Inputs from more distant locales are lost preferentially, suggesting a focusing of local inhibitory inputs as development proceeds.

While the pattern of inhibitory inputs seen by P36 to P40 was adult-like, the percentage of sites generating IPSCs continued to decrease after P41. The decline close to the cell bodies was not significant (37 ± 3% to 26 ± 4%; Figures 3E and 4A; *t*-test, *P *< 0.08; n = 31), but the three-fold decline at longer distances (>250 μm) was highly significant (14 ± 2% at P36 to P40 to 4 ± 1% at >P41; *t*-test, *P *< 0.0001). In mature animals, most sites generating IPSCs were located within 250 μm of the recorded neurons and almost all were within 500 μm. Local inhibitory inputs formed a largely radially symmetric distribution around the recorded neurons, and patchy patterns of long-range inhibitory inputs were robust at any age. We interpret these findings to indicate that inhibitory inputs transition through a period during which longer-range inhibitory connections are preferentially eliminated and that long-range inhibitory influences are sparse. Finally, although nearby inputs also decline during this period, they are more likely to be maintained as neurons attain the mature patterns of inhibitory inputs.

### Rearrangements of local excitatory synapses

The development of excitatory inputs was similar to that previously described [20] (Figure 3). At P20 to 22, excitatory inputs were sparse throughout the mapped region, with most excitatory post-synaptic currents (EPSCs) arising from within 250 μm of cell bodies (70 ± 8%, n = 7; Figures 3A and Figure 4B, Table 1). As development proceeded, the number of sites generating excitatory inputs increased dramatically, especially at more distant locales. Between P20 and P22 and P30 and P35, the percentage of sites generating EPSCs at locations greater than 1 mm from the recorded cells increased fivefold (from 6 ± 1% to 30 ± 7%, n = 25 cells; Figure 4B), a difference that is highly significant (*t*-test, *P *< 0.0002). After P41, the number of both local and longer-range inputs decreased; inputs from regions beyond 500 μm, although sparse, were frequently found in groups. This probably reflects the emergence of clustered horizontal connections (P30-P40 to >P41; 40.1 ± 3.9% to 29.3 ± 2.8% <250 μm; 30.5 ± 2.8% to 9.7 ± 0.9% <500 μm; n = 32; Figure 4B).

### Relationship between excitatory and inhibitory synaptic inputs

**Figure 5:**
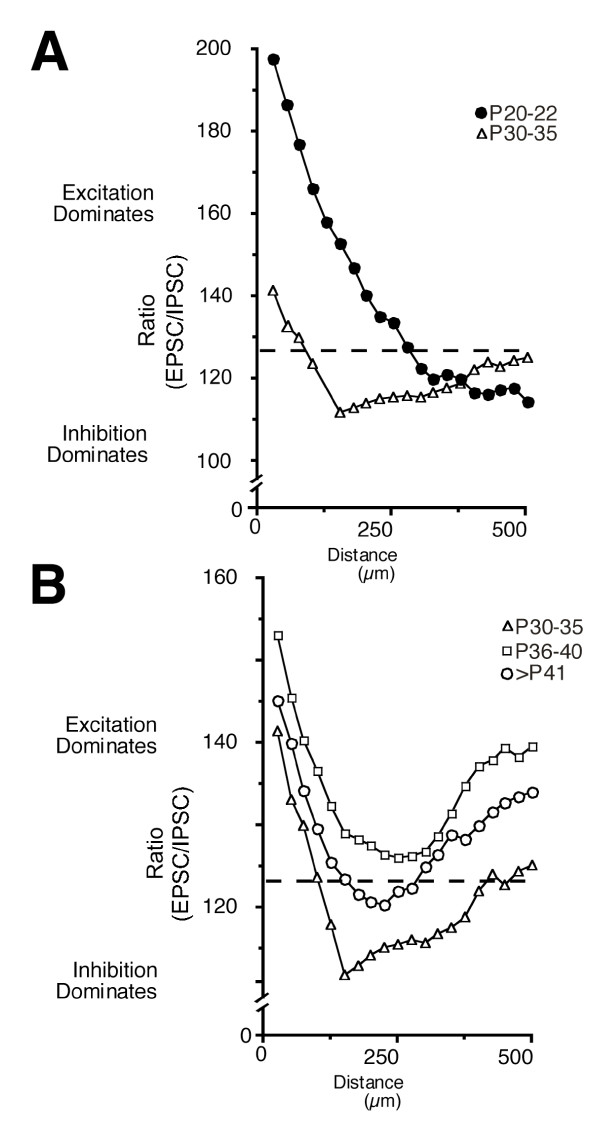
**Graph of the ratio of inhibition to excitation at increasing distance from the recorded neurons**. Each point in the graph represents the average ratio at that distance from all the neurons recorded in that age group (see Methods). **(A) **Comparison of the P20 to P22 age group (black circles) with the P30 to P35 age group (triangles). In the P20 to P22 age group, excitation dominates the region close to the recorded neurons out to about 300 μm; from there inhibition dominates. In the P30 to 35 age group, excitation dominates only out to about 150 μm from the recorded neurons; at greater distances inhibition dominates. **(B) **Comparison of the P30 to P35 age group (triangles), P36 to P40 age group (squares) and mature group (>P41; circles) indicates that the shape of the distributions of ratios is similar at these different ages. These plots demonstrate that the basic arrangement of inhibitory and excitatory connections remains stable. However, values that form the distribution vary significantly between each age group (ANOVA, *P *< 0.05), reflecting changes in the relative amount of excitation and inhibition within each age group.

To visualize the relationship between the patterns of EPSCs and IPSCs onto individual neurons, composite maps were constructed in which sites were coded as generating excitatory inputs only (red), both excitatory and inhibitory inputs (green), or inhibitory inputs only (blue) (Figure 3). The strength or number of inputs from each location is omitted from these plots. To quantitatively measure the relative amount of excitation or inhibition across our data set, we calculated the ratio of EPSCs to IPSCs for each map and then averaged these patterns together (Figure 5; see Methods). Because we have found previously that strength of connections correlates well with the number of events following a stimulus [20,21], we based our calculations on the number of evoked inputs detected at each site stimulated.

Between P20 and P22, excitatory inputs dominated the regions close to the recorded cells (<250 μm), and more distant locations were dominated by inhibitory inputs (Figure 3B). The ratio of inhibitory to excitatory inputs increased with increasing distance from the cell bodies; by 300 μm, inhibitory inputs numerically dominated excitatory inputs (Figure 5A). At these ages inputs from beyond 700 μm were rare (<10%); those present were primarily excitatory. These might correspond to the initial synaptic contacts of unbranched long-range horizontal projections already present at this age [8].

The relationships between inhibition and excitation undergo two major shifts between P20 and P22 and older ages. First, in the youngest animals a much larger area close to recorded neurons was dominated by excitatory inputs (275 μm versus 150 μm diameter; Figure 3B versus Figures 3D and 5A; X^2^, *P *< 0.05). Second, the region dominated by inhibitory inputs in younger animals (beyond approximately 300 mm from the cell body) becomes sparse with inputs but is dominated by excitation in older animals (Figure 3F versus Figures 3H and 5B; X^2^, *P *< 0.05).

By P30 to P35 the relationship between excitation and inhibition already resembles that of mature cells, although, as described above, more IPSCs than EPSCs are evoked within the mapped regions (Figures 3 and 5B). In fact inhibition dominates a larger annular region surrounding the cell body at this age (approximately 300 μm versus approximately 125 μm; Figures 3D and 5B). In the P36 to P40 age group inhibition does not dominate at any distance, indicating a relative increase in the prevalence of excitation. This is due not to an actual increase in the number of excitatory inputs, but rather to a decline in the number of inhibitory inputs at all distances (Figures 3F and 4). In the mature age group, the number of excitatory inputs declines, and inhibition again dominates an annular region surrounding the recorded neurons (Figures 3H and 5B). Thus, inhibitory inputs refine prior to the excitatory ones.

### Mechanism guiding remodeling of inhibitory connections

The rearrangements of inhibitory connections we observed could result from numerous mechanisms, but the similarities between the timing and extent of changes in the patterns of inhibitory and excitatory connections suggest the intriguing possibility that similar mechanisms underlie both phenomena. During development in the cortex, hippocampus, and auditory brainstem, GABA can act as an excitatory transmitter [19]. As our whole-cell patch clamp recording conditions quickly dialyze neurons and alter their native chloride concentration, we did not address the question of whether GABA is depolarizing during development. Others, however, have found that GABA application evokes excitatory currents in developing rat cortical neurons and causes influx of calcium [22-25]. Thus, the inhibitory inputs we observed may normally depolarize developing neurons, and it is reasonable to hypothesize that the rearrangements we observe could utilize correlation-based, activity-dependent mechanisms.

**Figure 6:**
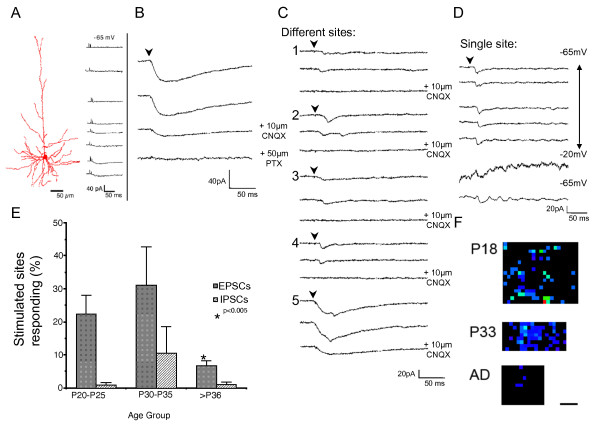
**Uncaging GABA generates excitatory inputs in brain slices from immature animals**. **(A) **Left: camera lucida reconstruction of a biocytin filled neuron in a coronal brain slice. Right: responses of neuron shown in A to uncaging of GABA at locations along its apical and basal dendrites. **(B) **Effects of picrotoxin (50 mM) and CNQX (10 mM) treatment on the response of a cell to uncaging of GABA close to the cell soma. **(C) **Effects of CNQX blockade on photostimulation-evoked responses from distant uncaging of GABA. Locations 1 to 4 are >200 μm from the location of the cell body of the recorded neuron. Location 5 is near to the neuron. CNQX blocks the synaptic response, but not the photostimulation-evoked GABA-mediated current. **(D) **The same locations stimulated a number of times at different holding potentials with uncaging of GABA in a neuron. **(E) **Graph of the percent of sites stimulated with uncaged GABA in tangential brain slices that generate a synaptic response at different ages. (P20 to P25, n = 11 cells, 348.5 ± 52.8 stimulated sites; P30 to 35, n = 12 cells, 316.6 ± 44.2; <P36, n = 15 cells, 280.5 ± 57.4). Error bars = standard error of the mean. **(F) **Examples of PSC maps evoked with uncaging of GABA. P20 map consists of 482 stimulated sites covering an area of 0.82 mm^2^. P34 map consists of 428 stimulated sites covering an area of 0.34 mm^2^. Mature map consists of 239 stimulated sites covering an area of 0.30 mm^2^. Scale bar = 250 μm.

To address this possibility, we conducted experiments using caged-GABA. We tested whether photo-uncaging of GABA could evoke excitatory synaptic currents in 38 recorded neurons from cortical slices. First we asked whether focal uncaging of GABA could evoke responses in our recording neurons. Photo-uncaging of GABA near to the recording electrode resulted in large slow currents that were blocked by picrotoxin but not CNQX or AP-5 (Figure 6A, B). Then, using a similar protocol to that used for uncaging glutamate, we uncaged GABA at an array of locations surrounding cortical neurons in tangential brain slices of ferret visual cortex. GABA uncaging resulted in synaptic currents that were reversibly blocked by CNQX application (Figure 6C, D). Thus, uncaging of GABA depolarized neurons past the spike threshold and resulted in an excitatory response in neurons strong enough to evoke excitatory synaptic currents in contacting neurons.

To test whether synaptic currents evoked by uncaging of GABA was developmentally regulated, we recorded from neurons at different developmental ages. When we recorded from neurons from immature brain slices (P22 to P25), we found numerous locations that gave rise to synaptic currents (Figure 6E). Between P30 and P35, when inhibitory currents evoked by glutamate uncaging have begun to refine, photostimulation maps generated with GABA uncaging continue to display numerous locations that evoke excitatory synaptic currents (Figure 6E). These finds suggest that GABA is still acting in brain slice as an excitatory transmitter at these times and that the general pattern of inputs evoked by GABA uncaging at young ages is similar to that seen for glutamate uncaging. More work will be needed to determine whether there are fine scale differences in the patterns of inputs generated by GABA versus glutamate uncaging. However, after P36 when inhibitory inputs have achieved their mature pattern after a period of rapid refinement, uncaging GABA results in few evoked excitatory synaptic currents (neurons, Figure 6E) and the pattern of inputs differs greatly from that seen with glutatmate uncaging. Thus, during the period that GABAergic inhibitory inputs are undergoing refinement, GABA uncaging can act to excite neuronal activity that evokes excitatory synaptic inputs.

## Discussion

Using a functional assay for the organization of synaptic inputs, we found that both inhibitory and excitatory synaptic currents were present as early as P20 in layers 2 and 3 of ferret visual cortex. Both excitatory and inhibitory inputs were initially sparse, but their numbers increased steadily to reach a maximum number and spatial extent by P30 to P35. The number and dispersion of sites generating inhibitory inputs quickly refined to the mature pattern, indicating that inhibitory circuits, like excitatory ones, undergo a period of remodeling during which some connections are functionally lost. The data also suggest that the refinement of inhibitory connections precedes the refinement of excitatory connections, but further work is needed to show this definitively. In the case of inhibitory connections, most of the loss is from sites distant from the neuronal cell body, implying that the region of inhibitory influences becomes increasingly focused as visual behavior begins. Finally, the timing of refinement of inhibitory inputs correlates with a switch in the activity of GABA from excitatory to inhibitory, suggesting a potential link between the ability of GABA to act as an excitatory neurotransmitter *in vitro *and changes in and the rapid refinement of inhibitory inputs observed.

We focused on understanding how inhibitory and excitatory inputs develop in the simplified ferret brain slice system. By making tangential slices through the superficial layers of visual cortex, we examined the development of these inputs onto pyramidal cells. By leaving intralaminar and local connections intact, but removing interlaminar connections, we could focus on the emergence of the pattern of inputs just within layers 2/3. The main sources of inhibition in layers 2/3 are basket and chandelier type neurons and inhibitory neuron axons are typically restricted to a single layer [26-28]. Therefore, our maps are likely to reflect the pattern of connections made by these two classes of neurons. Other prominent cells types, such as double bouquet cells, typically have arbors in layer 1 [27], and so their inputs are unlikely to be detected in our mapping approach. Thus, our data likely reflect the emergence and maturation of intralaminar connections between basket, chandelier and pyramidal neurons. Interestingly, our data provide evidence for both longer range and local inhibitory inputs, suggesting that connections necessary for both feedback models of orientation selectivity and lateral connectivity models may exist [29,30].

Our results are consistent with reports that indicate inhibitory connections are formed just before or as excitatory connections begin to develop [18,19,24,25]. Immunocytochemical examination of glutamate decarboxylase (GAD) and other markers of inhibitory neurons in ferret visual cortex indicate that they are present throughout the period examined in our studies [31,32], and anatomical studies of nonpyramidal neurons in the cat indicate that extensive axonal arbors are present at birth [33]. The data are also broadly consistent with other functional mapping experiments that have mapped the pattern of inhibitory connections in cortex [18]. However, by mapping both excitatory and inhibitory connections onto the same neurons during development, our data provide new information regarding how the patterns of excitatory and inhibitory inputs emerge as neurons mature. In addition, this study begins to examine potential mechanisms that could mediate the refinement of inhibitory connections using caged GABA. Taken together, our work demonstrates that the mature patterns of inhibitory and excitatory inputs onto single neurons emerge through processes that have similar steps, but have significant differences in timing, suggesting the mechanisms underlying the development of excitatory and inhibitory connections may rely on similar but distinct mechanisms.

### Loss of synaptic connections

The substantial reduction in the number of physiologically observed synaptic inputs seems, at first glance, inconsistent with the well-documented increase in the number of anatomically observed synaptic contacts during development [34-37]. Factors such as decreasing cell density, decreased probability of synaptic transmission, or changes in glutamate receptor composition that have been observed during development might contribute to the observed change in evoked inputs. However, it is also likely that electron-microscopic identification of synaptic contacts in developing tissue underestimates the number of functional contacts present. Several lines of evidence are consistent with this possibility. At the neuromuscular junction and in the frog tectum, for example, functional synaptic contacts can form rapidly without the obvious presence of morphologically identifiable pre- or postsynaptic elements [38,39]. In the developing lateral geniculate nucleus, functional synapses are easily detectable, but are difficult to identify using electron microscopy [40]. In the developing tectum, contacts often form transiently for short durations [41-43]. Based on this evidence, it is likely that the neuropil of the developing cortex is composed mostly of such immature synapses, which can form and break rapidly. The widespread distribution of synaptic vesicle proteins and release machinery in neonatal animals [44], compared with their exclusive localization in synaptic boutons in the adult, are consistent with the idea that developing synapses may be both transitory and difficult to discern morphologically. Consistent with this possibility, evoked inhibitory inputs strengthen as the mature pattern of connectivity emerges [18].

### Remodeling of inhibitory connections

Our experiments indicate that during development inhibitory connections in striate cortex undergo a sequence of events similar to that of excitatory connections: they are initially sparse, undergo a period of rapid elaboration, and finally refine to their adult pattern. In systems in which significant remodeling of excitatory projections occurs, neuronal activity has been implicated as a driving force in the process (reviewed in [45,46]). There are now numerous examples of remodeling of inhibitory inputs, and evidence for the role of both activity-dependent and -independent mechanisms in these events [3,19]. In our study the potential role of ketones, which may influence the response of immature neurons to GABA, was not examined [47]. However, the data indicated that a rapid refinement of inhibitory inputs occurs as the response of neurons in brain slice to uncaged GABA shifts from excitatory to inhibitory, suggesting a possible role for neuronal activity or neuronal metabolism in the change in patterns of inhibitory connections.

### Inhibitory connections and the emergence of response properties

Emergence of the adult pattern of inhibitory connections coincides closely with the appearance of the mature map of orientation selectivity [48,49]. Using intrinsic signal imaging and single-unit recordings, Chapman *et al*. [49] first resolved the orientation map between P30 and P33. This roughly corresponds with the reduction, between P35 and P40, in the percentage of sites giving rise to inhibitory inputs. Achievement of the adult pattern of functional long-range excitatory connections after P41 also coincides with the time that the map of orientation attains its adult strength. The correlation between the refinement of inhibitory and excitatory connections and the formation of the adult pattern of orientation-selective regions suggests a potential role of intrinsic circuitry in the generation of this pattern. In light of several studies that suggest that local circuits are involved in generating orientation selectivity [13], our results imply that there may be a relationship between the emergence of the map of orientation selectivity and the patterns of inhibitory and excitatory synaptic connectivity within the visual cortex.

## Methods

### Slice preparation

Slices were prepared using methods previously described [20,21]. Briefly, animals ranging in age from P20 to P60 were deeply anesthetized with Nembutal (100 mg/kg intraperitoneally). The brain was removed and visual cortex dissected in chilled sucrose artificial cerebrospinal fluid (Sucrose-ACSF, 248 mM sucrose, 5 mM KCl, 1.25 mM KH_2_PO_4_, 1.3 mM MgSO_4_, 2.1 mM CaCl_2_, 26 mM NaHCO_3_, 10 mM dextrose) [50]. Visual cortex was mounted and sectioned in 350-μm thick tangential slices using a Vibratome. To ensure that only layers 2 and 3 were studied, only the first section was used. Slices were maintained in an interface chamber heated to approximately 33°C. After approximately 45 minutes the sucrose-ACSF was removed and replaced with normal ACSF (NaCl-ACSF, 124 mM NaCl, 5 mM KCl, 1.25 mM KH_2_PO_4_, 1.3 mM MgSO_4_, 2.1 mM CaCl_2_, 26 mM NaHCO_3_, 10 mM dextrose). Slices were transferred to the recording chamber following at least one additional hour of recovery.

### Photostimulation

The method for scanning laser photostimulation has been described in detail elsewhere [21,51-53]. Briefly, a brain slice was placed in a small recording chamber with a glass coverslip bottom. The beam of a continuous wave UV argon laser was focused to a small spot (approximately 10 μm diameter) within the brain slice through an objective located beneath the brain slice. The laser was moved using a computer controlled x, y, z translation stage. ACSF containing 250 μm L-glutamic acid γ-(α-carboxy-2-nitrobenzyl) ester (CNB-caged glutamate; Invitrogen, Carlsbad, CA, USA) was continuously recirculated to maintain the slice while allowing stimulation of presynaptic neurons within the slice. Synaptic inputs onto single cells were monitored using conventional whole cell recording techniques [54]. Electrodes (5 to 9 MΩ) were filled with a standard internal solution (130 mM CsOH, 130 mM D-gluconic acid, 11 mM EGTA, 1 mM MgCl_2_, 1 mM CaCl_2_, 10 mM HEPES, 3 mM ATP, 1.8 mM GTP; pH = 7.2) containing 0.1 to 0.4% biocytin (Sigma-Aldrich, St. Louis, MO, USA). The response to each stimulus and the location of the stimulated site were recorded using custom software.

### Histology

After recording, slices were fixed with 4% paraformaldehyde, cryoprotected, and resectioned at 60 to 80 μm on a freezing microtome. Biocytin labeled cells were visualized by standard immunocytochemical staining techniques (Vectastain, antibody dilution 1:1,000-1:2,000) intensified with heavy metals [55]. To facilitate reconstruction of labeled neurons, usually only a single cell was recorded from each slice.

### Analysis

PSCs were counted throughout each 360-ms recording trace made following photolysis of caged glutamate, using software that discriminates PSCs by monitoring zero crossings in the derivative, the amplitude of the event, and length of the rise and decay time of the event. Inhibitory and excitatory events were discriminated by the sign of the rising phase of the derivative, and by an amplitude threshold. The results of the computer driven analysis were verified by a scientist blind to the position within the map of traces being analyzed. The program recorded the times at which events occurred within the trace, the number of events within the trace, and the location of the stimulus. A second program was then used to score whether a given location produced inhibitory responses only, a mixture of inhibitory and excitatory responses, or excitatory responses only. Maps from both programs were then combined to form a final validated excitatory and inhibitory map. Both sets of analyzed data were then discriminated so that only traces with events occurring within 70 ms of the stimulus were counted. This time was chosen based on the dynamics of the response to photo-uncaging, as the majority of photostimulation evoked spikes occur within this time [18,20,21]. Importantly, numerous studies and our own work have shown that photostimulation is unlikely to evoke polysynaptic currents. This is likely due to the poor time lock between uncaging and neuronal spiking, the small area of uncaging, sparse patterns of connectivity in cortex, poor time locking of action potentials generated by uncaging and theneed for multiple inputs to occur simultaneously [15,18,21,53,56,57]. Plots of the number of inhibitory or excitatory synaptic events and the sign of events within photostimulated regions were produced with Transform (Spyglass, Savoy IL, USA) to illustrate the locations generating inputs onto the recorded cell (Figures 1, 2, and 4). The percentage of sites responding within each age group were calculated from these plots with a custom program that counted the number of stimulated sites and responding sites within annuli of various diameters around the location of the cell body. The location of the recorded neuron was assumed to be the stimulated site evoking an action potential or in some cases the site of maximum evoked direct current. To validate this method, in some cases fluorescent latex microsphere injections were made into the brain slice and their location marked on the photostimulation map. The ratio of inhibition to excitation (Figures 4 and 5) was calculated within a 100 × 100 μm window passed over plots of the sign and number of synaptic events occurring within the first 70 ms of the stimulus. Within the window the total number of inhibitory and excitatory inputs was counted and their ratio determined. In windows that contained only excitatory or inhibitory events, the 0 in the numerator or denominator was replaced with a value of 0.01. The ratio values were then converted to a log scale, in which values greater than 2.0 indicate the presence of exclusively excitatory inputs, and values less than -2.0 indicate the presence of inhibitory inputs only. The range of log values was converted to 1 to 255, with a value of 128 representing a ratio of 1 (equal inhibition and excitation). The maps of the ratio of inhibition to excitation were compressed, so that angular information was disregarded to form the graph (Figure 5).

## Abbreviations

ACSF: artificial cerebrospinal fluid; EPSC: excitatory post-synaptic current; GABA: gamma-aminobutyric acid; IPSC: inhibitory post-synaptic current; P: postnatal day; PSC: post-synaptic current.

## Competing interests

The authors declare that he has no competing interests.

## Authors' contributions

MBD conducted the experiments and wrote the paper.

## References

[B1] Markram H Toledo-Rodriguez M Wang Y Gupta A Silberberg G Wu C Interneurons of the neocortical inhibitory system Nat Rev Neurosci 2004 5 793 807 10.1038/nrn1519 15378039

[B2] Dalva MB McClelland AC Kayser MS Cell adhesion molecules: signalling functions at the synapse Nat Rev Neurosci 2007 8 206 220 10.1038/nrn2075 17299456 PMC4756920

[B3] Huang ZJ Di Cristo G Ango F Development of GABA innervation in the cerebral and cerebellar cortices Nat Rev Neurosci 2007 8 673 686 10.1038/nrn2188 17704810

[B4] Hensch TK Critical period plasticity in local cortical circuits Nat Rev Neurosci 2005 6 877 888 10.1038/nrn1787 16261181

[B5] Luhmann HJ Martinez Millan L Singer W Development of horizontal intrinsic connections in cat striate cortex Exp Brain Res 1986 63 443 448 10.1007/BF00236865 3758263

[B6] Kisvarday ZF Eysel UT Functional and structural topography of horizontal inhibitory connections in cat visual cortex Eur J Neurosci 1993 5 1558 1572 10.1111/j.1460-9568.1993.tb00226.x 8124514

[B7] Albus K Wahle P The topography of tangential inhibitory connections in the postnatally developing and mature striate cortex of the cat Eur J Neurosci 1994 6 779 792 10.1111/j.1460-9568.1994.tb00989.x 7521250

[B8] Durack JC Katz LC Development of horizontal projections in layer 2/3 of ferret visual cortex Cereb Cortex 1996 6 178 183 10.1093/cercor/6.2.178 8670648

[B9] Ruthazer ES Stryker MP The role of activity in the development of long-range horizontal connections in area 17 of the ferret J Neurosci 1996 16 7253 7269 8929433 10.1523/JNEUROSCI.16-22-07253.1996 PMC6578943

[B10] Callaway EM Katz LC Effects of binocular deprivation on the development of clustered horizontal connections in cat striate cortex Proc Natl Acad Sci USA 1991 88 745 749 10.1073/pnas.88.3.745 1704130 PMC50890

[B11] Lowel S Singer W Selection of intrinsic horizontal connections in the visual cortex by correlated neuronal activity Science 1992 255 209 212 10.1126/science.1372754 1372754

[B12] Crair MC Ruthazer ES Gillespie DC Stryker MP Relationship between the ocular dominance and orientation maps in visual cortex of monocularly deprived cats Neuron 1997 19 307 318 10.1016/S0896-6273(00)80941-1 9292721

[B13] Chisum HJ Fitzpatrick D The contribution of vertical and horizontal connections to the receptive field center and surround in V1 Neural Netw 2004 17 681 693 10.1016/j.neunet.2004.05.002 15288892

[B14] Xu X Roby KD Callaway EM Mouse cortical inhibitory neuron type that coexpresses somatostatin and calretinin J Comp Neurol 2006 499 144 160 10.1002/cne.21101 16958092

[B15] Xu X Callaway EM Laminar specificity of functional input to distinct types of inhibitory cortical neurons J Neurosci 2009 29 70 85 10.1523/JNEUROSCI.4104-08.2009 19129386 PMC2656387

[B16] Kisvarday ZF Martin KA Whitteridge D Somogyi P Synaptic connections of intracellularly filled clutch cells: a type of small basket cell in the visual cortex of the cat J Comp Neurol 1985 241 111 137 10.1002/cne.902410202 4067011

[B17] Kisvarday ZF Gulyas A Beroukas D North JB Chubb IW Somogyi P Synapses, axonal and dendritic patterns of GABA-immunoreactive neurons in human cerebral cortex Brain 1990 113 793 812 10.1093/brain/113.3.793 2194628

[B18] Chen B Boukamel K Kao JP Roerig B Spatial distribution of inhibitory synaptic connections during development of ferret primary visual cortex Exp Brain Res 2005 160 496 509 10.1007/s00221-004-2029-4 15502991

[B19] Ben-Ari Y Excitatory actions of gaba during development: the nature of the nurture Nat Rev Neurosci 2002 3 728 739 10.1038/nrn920 12209121

[B20] Dalva MB Katz LC Rearrangements of synaptic connections in visual cortex revealed by laser photostimulation Science 1994 265 255 258 10.1126/science.7912852 7912852

[B21] Katz LC Dalva MB Scanning laser photostimulation: a new approach for analyzing brain circuits J Neurosci Methods 1994 54 205 218 10.1016/0165-0270(94)90194-5 7869753

[B22] Yuste R Katz LC Control of postsynaptic Ca2+ influx in developing neocortex by excitatory and inhibitory neurotransmitters Neuron 1991 6 333 344 10.1016/0896-6273(91)90243-S 1672071

[B23] Ben-Ari Y Tseeb V Raggozzino D Khazipov R Gaiarsa JL gamma-Aminobutyric acid (GABA): a fast excitatory transmitter which may regulate the development of hippocampal neurones in early postnatal life Prog Brain Res 1994 102 261 273 full_text 7800817 10.1016/S0079-6123(08)60545-2

[B24] LoTurco JJ Owens DF Heath MJ Davis MB Kriegstein AR GABA and glutamate depolarize cortical progenitor cells and inhibit DNA synthesis Neuron 1995 15 1287 1298 10.1016/0896-6273(95)90008-X 8845153

[B25] Owens DF Boyce LH Davis MB Kriegstein AR Excitatory GABA responses in embryonic and neonatal cortical slices demonstrated by gramicidin perforated-patch recordings and calcium imaging J Neurosci 1996 16 6414 6423 8815920 10.1523/JNEUROSCI.16-20-06414.1996 PMC6578913

[B26] Tamas G Lorincz A Simon A Szabadics J Identified sources and targets of slow inhibition in the neocortex Science 2003 299 1902 1905 10.1126/science.1082053 12649485

[B27] Karube F Kubota Y Kawaguchi Y Axon branching and synaptic bouton phenotypes in GABAergic nonpyramidal cell subtypes J Neurosci 2004 24 2853 2865 10.1523/JNEUROSCI.4814-03.2004 15044524 PMC6729850

[B28] Szabadics J Varga C Molnar G Olah S Barzo P Tamas G Excitatory effect of GABAergic axo-axonic cells in cortical microcircuits Science 2006 311 233 235 10.1126/science.1121325 16410524

[B29] Ben-Yishai R Bar-Or RL Sompolinsky H Theory of orientation tuning in visual cortex Proc Natl Acad Sci USA 1995 92 3844 3848 10.1073/pnas.92.9.3844 7731993 PMC42058

[B30] Priebe NJ Ferster D Inhibition, spike threshold, and stimulus selectivity in primary visual cortex Neuron 2008 57 482 497 10.1016/j.neuron.2008.02.005 18304479

[B31] Gao WJ Newman DE Wormington AB Pallas SL Development of inhibitory circuitry in visual and auditory cortex of postnatal ferrets: immunocytochemical localization of GABAergic neurons J Comp Neurol 1999 409 261 273 10.1002/(SICI)1096-9861(19990628)409:2<261::AID-CNE7>3.0.CO;2-R 10379919

[B32] Gao WJ Wormington AB Newman DE Pallas SL Development of inhibitory circuitry in visual and auditory cortex of postnatal ferrets: immunocytochemical localization of calbindin- and parvalbumin-containing neurons J Comp Neurol 2000 422 140 157 10.1002/(SICI)1096-9861(20000619)422:1<140::AID-CNE9>3.0.CO;2-0 10842223

[B33] Meyer G Ferres-Torres R Postnatal maturation of nonpyramidal neurons in the visual cortex of the cat J Comp Neurol 1984 228 226 244 10.1002/cne.902280209 6480914

[B34] Rakic P Prenatal genesis of connections subserving ocular dominance in the rhesus monkey Nature 1976 261 467 471 10.1038/261467a0 819835

[B35] Blue ME Parnavelas JG The formation and maturation of synapses in the visual cortex of the rat. I. Qualitative analysis J Neurocytol 1983 12 599 616 10.1007/BF01181526 6619906

[B36] Blue ME Parnavelas JG The formation and maturation of synapses in the visual cortex of the rat. II. Quantitative analysis J Neurocytol 1983 12 697 712 10.1007/BF01181531 6619907

[B37] Bourgeois JP Jastreboff PJ Rakic P Synaptogenesis in visual cortex of normal and preterm monkeys: evidence for intrinsic regulation of synaptic overproduction Proc Natl Acad Sci USA 1989 86 4297 4301 10.1073/pnas.86.11.4297 2726773 PMC287439

[B38] Dan Y Poo MM Retrograde interactions during formation and elimination of neuromuscular synapses Curr Opin Neurobiol 1994 4 95 100 10.1016/0959-4388(94)90037-X 8173331

[B39] Poo MM Activity-dependent modulation of developing neuromuscular synapses Adv Second Messenger Phosphoprotein Res 1994 29 521 527 7848730 10.1016/s1040-7952(06)80033-9

[B40] Campbell G Shatz CJ Synapses formed by identified retinogeniculate axons during the segregation of eye input J Neurosci 1992 12 1847 1858 1578274 10.1523/JNEUROSCI.12-05-01847.1992 PMC6575897

[B41] Wu G Malinow R Cline HT Maturation of a central glutamatergic synapse Science 1996 274 972 976 10.1126/science.274.5289.972 8875937

[B42] Cline HT Dendritic arbor development and synaptogenesis Curr Opin Neurobiol 2001 11 118 126 10.1016/S0959-4388(00)00182-3 11179881

[B43] Aizenman CD Cline HT Enhanced visual activity in vivo forms nascent synapses in the developing retinotectal projection J Neurophysiol 2007 97 2949 2957 10.1152/jn.00452.2006 17267761

[B44] Stettler O Moya KL Zahraoui A Tavitian B Developmental changes in the localization of the synaptic vesicle protein rab3A in rat brain Neuroscience 1994 62 587 600 10.1016/0306-4522(94)90391-3 7830899

[B45] Goodman CS Shatz CJ Developmental mechanisms that generate precise patterns of neuronal connectivity Cell 1993 72 Suppl 77 98 10.1016/S0092-8674(05)80030-3 8428376

[B46] Katz LC Shatz CJ Synaptic activity and the construction of cortical circuits Science 1996 274 1133 1138 10.1126/science.274.5290.1133 8895456

[B47] Rheims S Holmgren CD Chazal G Mulder J Harkany T Zilberter T Zilberter Y GABA action in immature neocortical neurons directly depends on the availability of ketone bodies J Neurochem 2009 110 1330 1338 10.1111/j.1471-4159.2009.06230.x 19558450

[B48] Chapman B Stryker MP Development of orientation selectivity in ferret visual cortex and effects of deprivation J Neurosci 1993 13 5251 5262 8254372 10.1523/JNEUROSCI.13-12-05251.1993 PMC6576418

[B49] Chapman B Stryker MP Bonhoeffer T Development of orientation preference maps in ferret primary visual cortex J Neurosci 1996 16 6443 6453 8815923 10.1523/JNEUROSCI.16-20-06443.1996 PMC2669086

[B50] Aghajanian GK Rasmussen K Intracellular studies in the facial nucleus illustrating a simple new method for obtaining viable motoneurons in adult rat brain slices Synapse 1989 3 331 338 10.1002/syn.890030406 2740992

[B51] Briggs F Callaway EM Layer-specific input to distinct cell types in layer 6 of monkey primary visual cortex J Neurosci 2001 21 3600 3608 11331389 10.1523/JNEUROSCI.21-10-03600.2001 PMC1820845

[B52] Schubert D Staiger JF Cho N Kotter R Zilles K Luhmann HJ Layer-specific intracolumnar and transcolumnar functional connectivity of layer V pyramidal cells in rat barrel cortex J Neurosci 2001 21 3580 3592 11331387 10.1523/JNEUROSCI.21-10-03580.2001 PMC6762473

[B53] Callaway EM Cell type specificity of local cortical connections J Neurocytol 2002 31 231 237 10.1023/A:1024165824469 12815242

[B54] Blanton MG Lo Turco JJ Kriegstein AR Whole cell recording from neurons in slices of reptilian and mammalian cerebral cortex J Neurosci Methods 1989 30 203 210 10.1016/0165-0270(89)90131-3 2607782

[B55] Adams JC Heavy metal intensification of DAB-based HRP reaction product J Histochem Cytochem 1981 29 775 7252134 10.1177/29.6.7252134

[B56] Shepherd GM Svoboda K Laminar and columnar organization of ascending excitatory projections to layer 2/3 pyramidal neurons in rat barrel cortex J Neurosci 2005 25 5670 5679 10.1523/JNEUROSCI.1173-05.2005 15958733 PMC6724876

[B57] Yoshimura Y Dantzker JL Callaway EM Excitatory cortical neurons form fine-scale functional networks Nature 2005 433 868 873 10.1038/nature03252 15729343

